# Myxomatous cause of multiple intracranial aneurysms and cognitive decline: a case report

**DOI:** 10.1186/s43044-024-00559-2

**Published:** 2024-09-16

**Authors:** Sudipta Mondal, Prabhu Selvaraj, Asish Vijayaraghavan, Viswanadh S. V. G. Kalaparti, Deepti Narasimhaiah

**Affiliations:** 1 Department of Cardiology, Sree Chitra Tirunal Institute for Medical Sciences and Technology, Thiruvananthapuram, India; 2 Department of Neurology, Sree Chitra Tirunal Institute for Medical Sciences and Technology, Thiruvananthapuram, India; 3 Department of Imaging Science and Intervention Radiology, Sree Chitra Tirunal Institute for Medical Sciences and Technology, Thiruvananthapuram, India; 4 Department of Pathology, Sree Chitra Tirunal Institute for Medical Sciences and Technology, Thiruvananthapuram, India

**Keywords:** Myxomatous, Intracranial aneurysms, Myxoma, Atrial myxoma

## Abstract

**Background:**

The occurrence of cerebral aneurysm in a case of cardiac myxoma is rare with less than 60 cases reported worldwide. The course of management is still debatable given its rarity. We present a case of multiple intracranial aneurysms secondary to atrial myxoma in a young lady with a brief review of the literature.

**Case presentation**

A young lady in her late 30s with a history of right middle cerebral artery territory stroke eight years ago presented with gradually progressive symptoms in the form of holocranial headache, inattention and forgetfulness for the last few years. On neuroimaging, she was found to have multi-territorial lacunar infarcts and multiple intracranial artery aneurysms which was confirmed with a digital subtraction angiogram. A cardiac evaluation revealed a left atrial myxoma. The aetiology of subcortical cognitive decline and intracranial aneurysms was attributed to the myxoma with secondary myxomatous embolism. Other secondary causes were ruled out. She is being followed up medically after resection of the myxoma.

**Conclusion:**

Intracranial aneurysms are rare complications of cardiac myxoma which may present before, concurrent or many years after diagnosis of the myxoma. Nonspecific neurological complaints occasionally are the ominous signs of intracranial aneurysms which mandate a low threshold for neuroimaging in a case of cardiac myxoma. Given the absence of definitive risk factors and unclear natural history, clinical and radiological follow-ups are critical.

**Learning Points**

- Intracranial aneurysms are rare complications of cardiac myxoma that may present before, concurrent or many years after diagnosis of the myxoma.
- Special attention must be given to nonspecific neurological complaints with a low threshold for neuroimaging in those with a prior history of cardiac myxoma.
- Given the absence of definitive risk factors and unclear natural history, clinical and radiological follow-up including conventional angiography and/or magnetic resonance imaging is critical.

## Background

Cardiac myxoma is the most common primary benign tumour of the heart, comprising approximately half of all cases. It has an incidence of 0.5–1 case per million individuals. The left atrium is most frequently affected in atrial myxoma, and echocardiography is the diagnostic modality of choice [1]. Myxomas are biologically benign with good long-term prognosis, but they are functionally malignant as embolism occurs in 30% to 40% of patients [2]. Although neurological complications are well-documented, the occurrence of cerebral aneurysms is rare, with fewer than 60 cases reported worldwide [3]. The possible management course of these aneurysms is still debatable given its rarity. We present a case of multiple intracranial aneurysms secondary to atrial myxoma in a young lady. Moreover, we present a brief review of the pathogenesis, natural history and management of myxomatous aneurysms.

### Case presentation

A 37-year-old female presented with a history of mild headache, forgetfulness and cognitive decline for over 6 years which was worsening gradually. Eight years prior, the patient experienced an acute onset of left-sided weakness, facial deviation and slurred speech, which nearly completely resolved without medical intervention.

On examination, she had spastic dysarthria, left upper motor neuron facial palsy, left hemiparesis and grade 2 spasticity of lower limbs. Neuropsychology evaluation revealed a subcortical pattern of cognitive impairment with mild executive dysfunction and impairment of recent verbal memory.

**Fig. 1:**
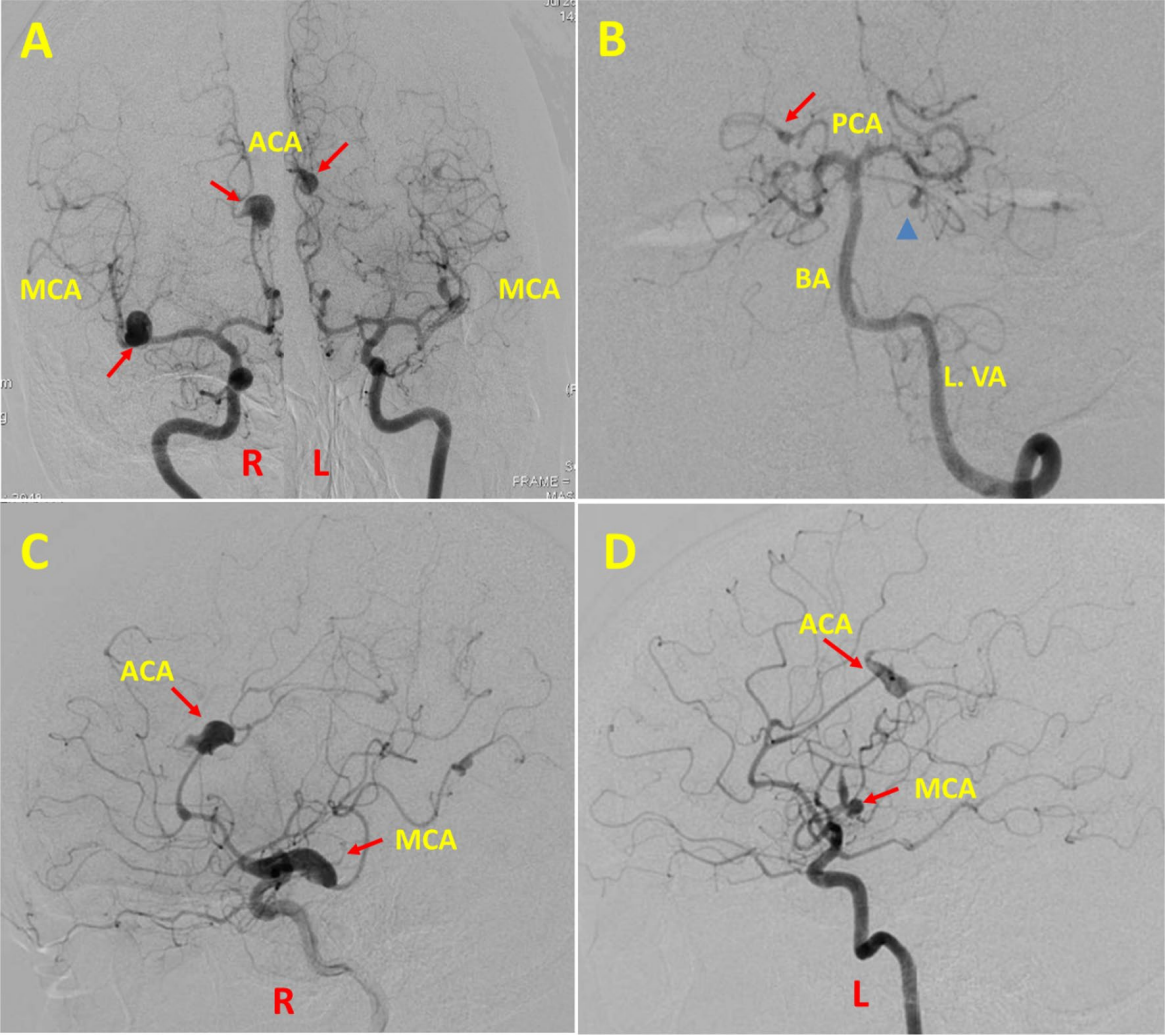
**A** Right and left ICA anteroposterior view angiograms show the fusiform aneurysm at the right middle cerebral artery bifurcation, along the pericallosal branch of the right anterior cerebral artery, along the distal superior division of the left middle cerebral and the left pericallosal artery. **B** Selective left vertebral artery angiogram in AP view shows a fusiform aneurysm along the right distal posterior cerebral artery (from the P4 segment, arrow) and a saccular aneurysm along the left proximal superior cerebellar artery (arrowhead). **C** Right ICA lateral angiogram shows the fusiform aneurysms at the right middle cerebral artery bifurcation extending along the inferior and along the right pericallosal branch of the anterior cerebral artery. Multiple smaller aneurysms at the distal anterior and middle cerebral artery branches are also seen. **D** Left ICA lateral angiogram shows the fusiform aneurysms along the distal superior division of the left middle cerebral artery and at the distal left pericallosal artery, a branch of the left anterior cerebral artery. R: right; L: left; ICA: internal carotid artery; ACA: anterior cerebral artery; MCA: middle cerebral artery; PCA: posterior cerebral artery; BA: basilar artery; L. VA: vertebral artery

**Fig. 2:**
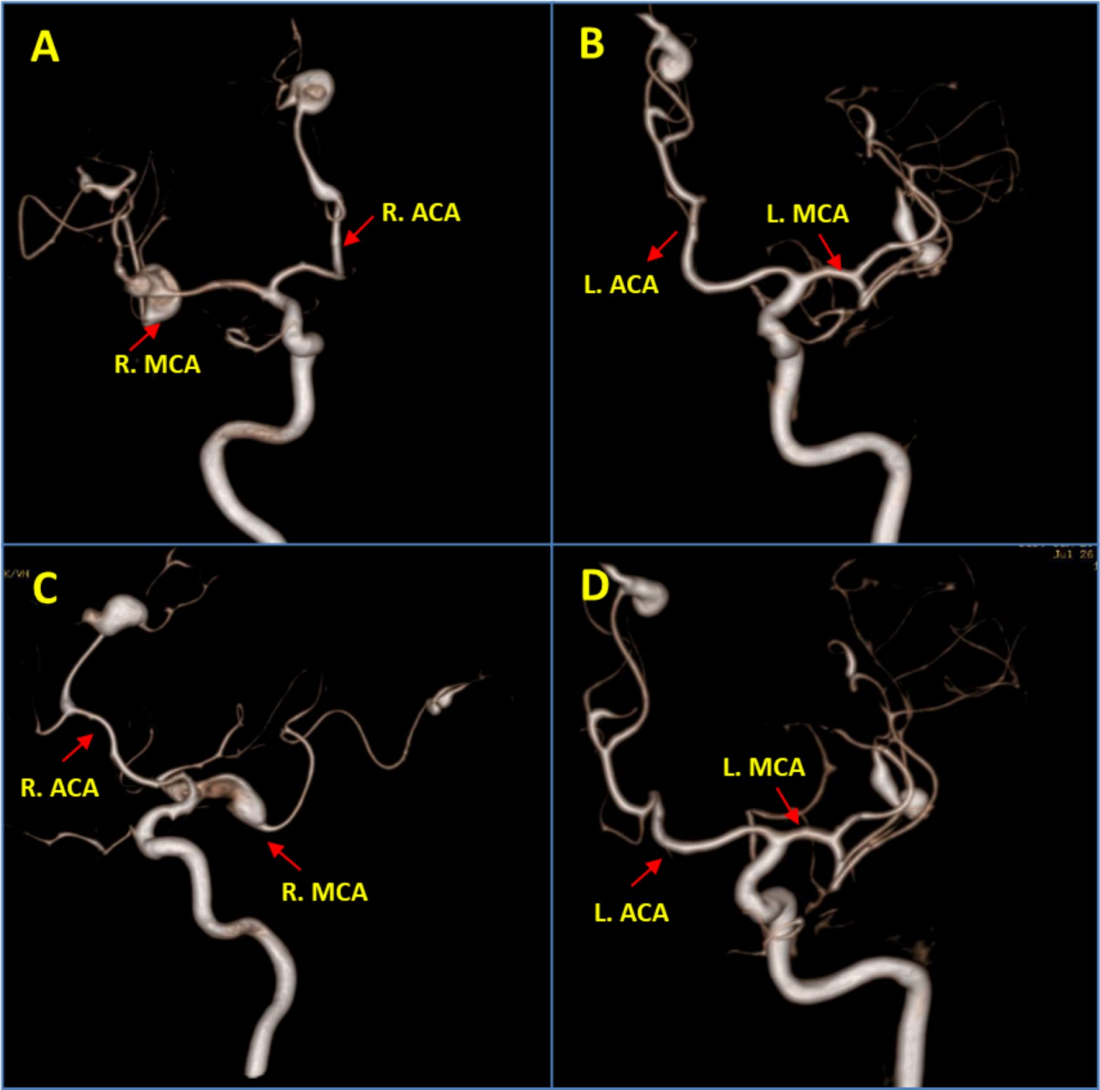
Volume-rendered images generated from the selective right ICA angiogram (**A**, **C**) and the left ICA angiogram (**B**, **D**) in anterior oblique views show the fusiform aneurysms along the right MCA bifurcation, the distal superior division of the left MCA and along the distal aspect of pericallosal branches of bilateral anterior cerebral arteries. R: right; L: left; ICA: internal carotid artery; ACA: anterior cerebral artery; MCA: middle cerebral artery

**Fig. 3:**
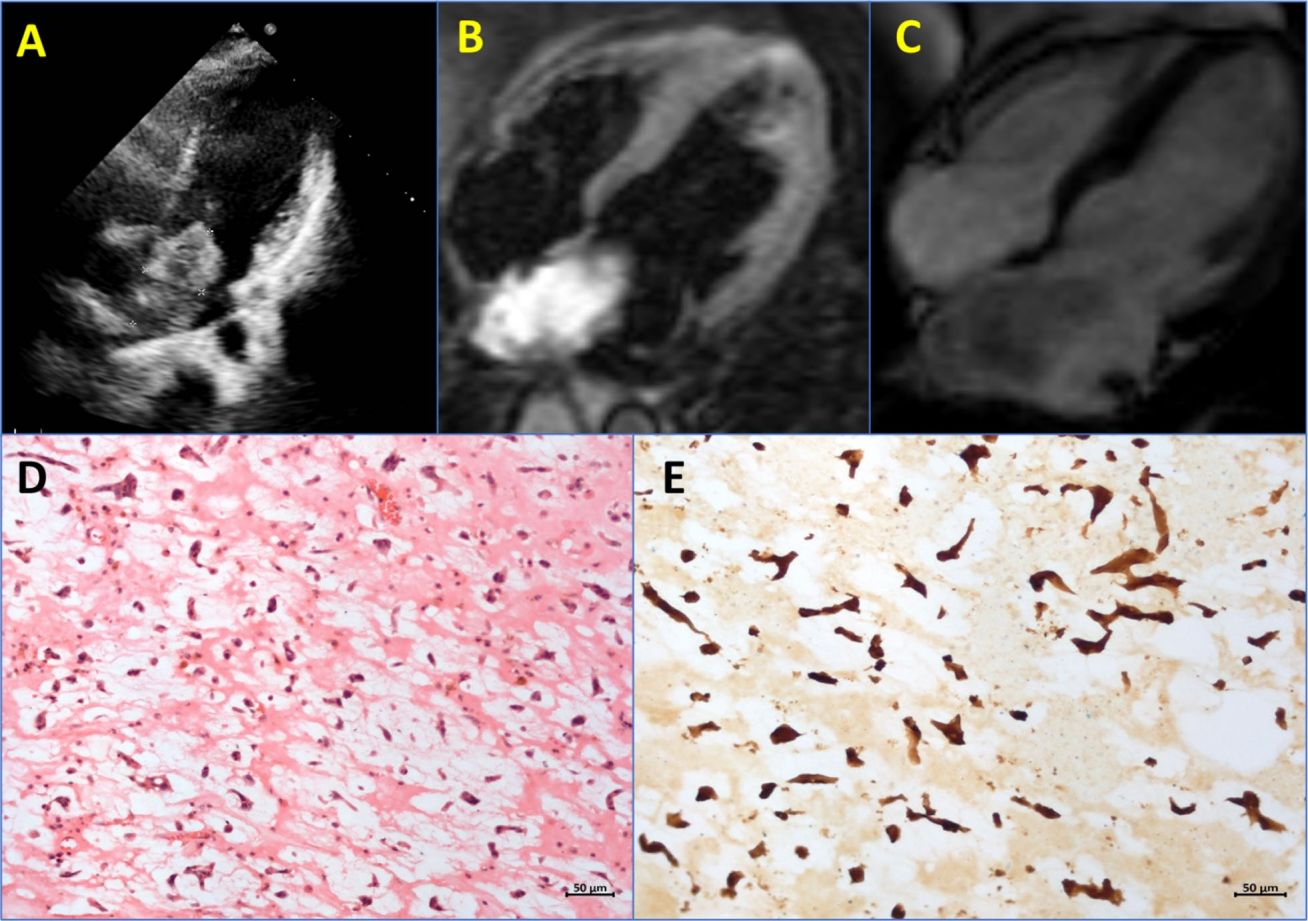
**A** Apical 4-chamber 2D echocardiogram showing heterogeneous well-circumscribed left atrial mass attached to the interatrial septum, **B** A mass lesion was seen in the left atrium, appearing hyperintense on black blood and fluid sensitive (T2/STIR) and hypointense on bSSPF **C** images, **D**, **E** Histopathology of myxoma with myxoma cells in myxoid stroma (**D**) expressing calretinin (**E**). [D: Haematoxylin and Eosin, E: Immunoperoxidase, Original magnification = Scale bar]. STIR: short tau inversion recovery; bSSPF: balanced steady-state free precession.

Blood routine investigations including complete blood count, and renal and liver function tests were normal. The autoimmune and vasculitic profile was negative. Vitamin B12, homocysteine level and thyroid function test were normal. The workup for syphilis was negative. Magnetic resonance imaging (MRI) brain showed chronic lacunar infarcts involving bilateral basal ganglia, left midbrain and cerebelli. Magnetic resonance angiogram (Time of flight) of the brain showed multiple intracranial aneurysms involving anterior and posterior cerebral circulation. Digital subtraction angiogram (DSA) was planned for the characterisation of the aneurysms which showed multiple fusiform and saccular aneurysms involving right MCA bifurcation, right MCA and ACA distal branches, left MCA superior division, bilateral ACA involving pericallosal arteries at the bifurcation points, the parieto-occipital branch of the right posterior cerebral artery (PCA) and along the left superior cerebellar artery (Figs. 1A–D, 2A–D). 2D echocardiogram showed a 46 × 22 mm mass in the left atrium (Fig. 3A). Cardiac MRI was suggestive of left atrial myxoma attached to interatrial septum prolapsing across the mitral valve (Fig. 3B, C). The aetiology of cognitive decline and intracranial aneurysms was attributed to left atrial myxoma with secondary myxomatous embolism.

She underwent a surgical excision of myxoma which on histopathological examination showed clusters and cords of myxoma cells set in a myxoid stroma (Fig. 3D). Furthermore, the myxoma cells were positive for calretinin immunostain (Fig. 3E).

## Discussion

Cardiac myxoma is a tumour of mesenchymal origin comprising almost half of all primary cardiac tumours [4]. As many as 10% of patients with atrial myxoma remain asymptomatic. They usually present with systemic embolism, intracardiac obstruction, constitutional symptoms and rarely with arrhythmia or acute coronary syndrome (Table 1) [5, 6]. Acute ischaemic stroke is the most common neurological sequel secondary to tumour embolism, and others include intracranial aneurysms, vascular malformation and parenchymal metastases [7]. In a recent systematic review of 55 patients with atrial myxomas and intracranial aneurysms described to date, the majority were females (65%) and young (age < 60 years, 86%) [3, 8]. Aneurysms were detected before, during or even 25 years after the diagnosis/resection of the atrial mass [3].

**Table 1:** Comparison of the index case with existing reported cases (summarised from a systematic review by Chojdak-Łukasiewicz J et al. [3])

Clinical presentation	Location of the aneurysm
Vascular incidents—TIA, Stroke (36%)	Multiple territory (31%)
Headache (27%)	MCA (29%)
Seizures (16%)	MCA + ACA (13%)
Vertigo/dizziness (15%)	MCA + PCA (9%)
Loss of consciousness (7%)	PCA (9%)
Subarachnoid haemorrhage (6%)	ACA + PCA (6%)
Asymptomatic (4%)	BA (4%)

Management	Cardiac myxoma surgery
Conservative (60%)	100%
Chemotherapy (6%)	
Stereotactic radiosurgery (2%)	
Radiotherapy (2%)	
Resection of aneurysm/bypass/aneurysm clipping/coiling (27%)	

Index case: Stroke, Headache, cognitive decline, multiple intracranial territory involvement, aneurysm managed conservatively, cardiac myxoma resected

TIA: transient ischaemic attack, MCA: middle cerebral artery, ACA: anterior cerebral artery, PCA: posterior cerebral artery, BA: basilar artery

The pathogenesis of aneurysm formation is not well defined, and several mechanisms have been postulated. Firstly, tumour cell infiltration of the vessel wall via vasa vasorum leads to vessel wall destruction. However, the relative lack of vasa vasorum in the intracranial arteries in human autopsy studies contradicts the proposal [9]. Secondly, occlusion by tumour emboli causes subsequent scarring and pseudoaneurysm [10]. Thirdly, breach of the internal elastic lamina of vessels by direct invasion by the tumour cells, subsequent IL-6-mediated inflammation and damage to the arterial wall by excess matrix metalloproteinases leading to subsequent aneurysm formation [11, 12]. This is supported by the histological examination in a few case reports [12].

The natural history of the aneurysm is not well defined. Few reports have shown the stability of the lesions over many years, or even regression with excision of myxoma [13, 14]. In patients with atrial myxoma and intracranial aneurysms, the most common manifestation was vascular incidents (25%) in the form of transient ischaemic attacks or strokes secondary to embolism and aneurysms were mostly detected coincidentally [3]. Patients with multiple intracranial aneurysms may be asymptomatic or experience nonspecific headaches which can be predictive as seen in our case [3]. Seizures and dizziness have also been reported secondary to microbleeding from aneurysms. Subarachnoid or intracerebral bleeds were very rare.

Magnetic resonance imaging and computed tomography can show infarcts, haemorrhage or aneurysms. T1-weighted MRI images may show aneurysmal segment and may show enhancement either due to a slow flow phenomenon or enhancement of the tumour cells/myxoid matrix within the vessel wall [15, 16]. Conventional angiogram may be planned for the characterisation of the aneurysms which are commonly multiple and fusiform located more peripherally. Anterior and middle cerebral arteries are the most common arteries involved. Extracerebral artery involvement is very rare due to the avidity of myxoma cells for cerebral blood vessels [8] .

Cognitive disturbance is an uncommon presentation in atrial myxomas which can be due to recurrent infarcts or bleeding from aneurysms. Our patient presented with cognitive decline secondary to recurrent subcortical infarcts caused by myxomatous embolism which could have been averted had she undergone evaluation for her stroke earlier. Digital subtraction angiogram showed multiple aneurysms at distal branches which were predominantly fusiform, the aetiology of which can be attributed to myxoma in the absence of other risk factors or genetic morphology for aneurysms.

The atrial myxoma should be excised as soon as possible after the diagnosis to prevent further complications. During surgery, three important points are to be ensured to prevent recurrence and future complications: (1) removal of the multi-focal tumour; (2) removal of appendages when and where required; (3) avoidance of tumour fragmentation and embolization [17].

Currently, there is a lack of specific guidelines for the treatment of aneurysms caused by cardiac myxomas. However, a watchful clinical and radiological follow-up is recommended [15, 18]. A lot of therapeutic methods, ranging from endovascular methods, surgery, chemotherapy, radiation or a combination of these, are available. By default, endovascular treatment is not recommended because they are multiple, distally located, fusiform and without neck. Only enlarged or ruptured aneurysms may require invasive management and must be evaluated for endovascular or neurosurgical intervention [19]. Open surgical management is required for a lesion causing mass effect or in a single saccular aneurysm not amenable to percutaneous interventions.

In recent years, chemotherapy with doxorubicin or a combination of etoposide and carboplatin was useful in some cases in conjunction with surgical excision of myxoma [20, 21]. Chemotherapy may prevent aneurysm growth [22]. Low-dose radiation in combination with chemotherapy has been reported as an effective method for the degradation of metastasis [23]. A new option of frameless stereotactic radiosurgery (SRT) has emerged less invasive and less toxic alternative [24].

Despite the presence of multiple, small aneurysms, the patient's lack of serious aneurysm-related symptoms allowed for medical follow-up following the atrial myxoma excision. Nevertheless, close follow-up was recommended. The patient remained asymptomatic and showed no signs of neurological decline. Interval imaging was deemed unnecessary in the absence of symptoms.

## Conclusion

Intracranial aneurysms are rare complications of cardiac myxoma that may present before, concurrent or many years after diagnosis of the myxoma. Special attention must be given to nonspecific neurological complaints with a low threshold for neuroimaging in those with a prior history of cardiac myxoma. Given the absence of definitive risk factors and unclear natural history, clinical and radiological follow-up including conventional angiography and/or magnetic resonance imaging is critical.

## Data Availability

No new data were generated or analysed supporting this research.
